# Virus-induced host genomic remodeling dysregulates gene expression, triggering tumorigenesis

**DOI:** 10.3389/fcimb.2024.1359766

**Published:** 2024-03-20

**Authors:** Weixia Dong, Huiqin Wang, Menghui Li, Ping Li, Shaoping Ji

**Affiliations:** ^1^ Department of Basic Medicine, Zhengzhou Shuqing Medical College, Zhengzhou, Henan, China; ^2^ Department of Biochemistry and Molecular Biology, Medical School, Henan University, Kaifeng, Henan, China

**Keywords:** virus, genome, epigenetic modification, DNA remodeling, tumorigenesis

## Abstract

Virus-induced genomic remodeling and altered gene expression contribute significantly to cancer development. Some oncogenic viruses such as Human papillomavirus (HPV) specifically trigger certain cancers by integrating into the host’s DNA, disrupting gene regulation linked to cell growth and migration. The effect can be through direct integration of viral genomes into the host genome or through indirect modulation of host cell pathways/proteins by viral proteins. Viral proteins also disrupt key cellular processes like apoptosis and DNA repair by interacting with host molecules, affecting signaling pathways. These disruptions lead to mutation accumulation and tumorigenesis. This review focuses on recent studies exploring virus-mediated genomic structure, altered gene expression, and epigenetic modifications in tumorigenesis.

## Introduction

Viruses can modify and affect host gene expression, particularly against the backdrop of tumorigenesis. Certain viruses have been identified as etiological factors in the development of various types of cancer (Haley et al., 2019; Mui et al., 2019; Hatano et al., 2021). By manipulating host gene expression, these oncogenic viruses can disrupt normal cellular processes, promote uncontrolled cell proliferation, and contribute to the formation of tumors (Krump and You, 2018). This review explores and analyzes the mechanisms through which viruses modify and influence host gene expression in tumorigenesis.

One of the primary ways viruses contribute to tumorigenesis is through the integration of the virus own genetic material into the host genome (Pinatti et al., 2018). This integration can occur near or within host genes, leading to alterations in their expression (Milavetz and Balakrishnan, 2015; Pinatti et al., 2018). For example, human papillomavirus (HPV) is a known oncogenic virus that integrates its DNA into the host genome. Intriguingly, the expression of viral oncoproteins, such as E6 and E7, they triggered hypermethylation of tumor suppressor genes and hypomethylation of proto-oncogenes, leads to the inactivation of tumor suppressor genes, such as p53 and pRb. The inactivation of these genes disrupts normal cell cycle regulation and promotes uncontrolled cell growth, contributing to the development of cervical and other HPV-associated cancers (Jones et al., 1997; Thomas et al., 1999).

**Figure 1:**
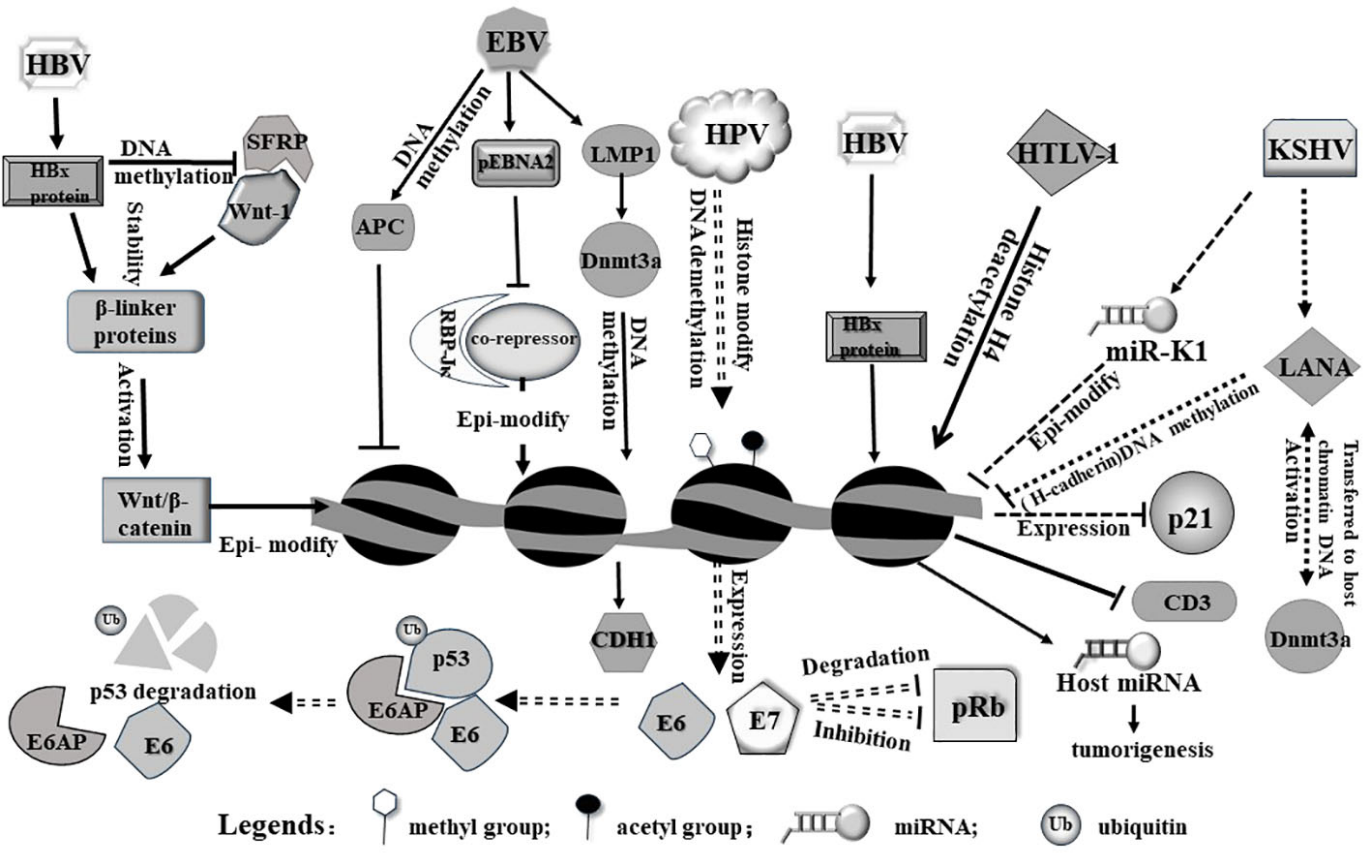
Viruses induce epigenetic modifications in the host cell primarily on genome via diverse pathways. HBx protein from HBV can release host Wnt-1 from SFRP/Wnt-1 complex. Free Wnt-1 cooperates with HBV to stabilize and activate Wnt/β-catenin, leading to epigenetic modification of the host genome. On the other hand, HBx protein alone can induce histone modification (including methylation and acetylation) and miRNA expression from the host genome. As a result, these modifications may trigger tumorigenesis. pEbNA2 from EBV represses association between RBP-Jκ and a co-repressor, modulating epigenetic signatures. In addition, LMP1 and APC encoded by EBV can modulate gene expression through genomic DNA methylation. HPV integrates into genome and activates expression E6 and E7, both of which act as an oncogene to induce p53 degradation or inhibit pRb. HTLV-1 virus induces and alters acetylation level of genome and affects relevant gene expression. In addition, viral microRNA from KSHV can epigenetically modulate host genome and alter host gene expression, such as inhibiting p21 expression.

In addition to direct integration, viruses can also indirectly modulate host gene expression by dysregulating cellular signaling pathways. Oncogenic viruses often express viral proteins that interact with host signaling molecules, leading to aberrant activation or inhibition of signaling cascades. This dysregulation can impact the expression of the downstream genes involved in tumorigenesis (Krump and You, 2018). For example, hepatitis B virus (HBV) produces the viral protein HBx, which can interact with multiple signaling pathways, including the Wnt/β-catenin pathway, mainly because it enhances the stabilization of cytoplasmic β-linker protein. HBX induces an increased expression of DNA methyltransferase 1 (DNMT1) and DNMT3A proteins toward the promoter region of secretory frizzled-related protein (SFRP), resulting in high methylation of the *SFRP* promoter and reduced protein expression. However, SFRP is a potent antagonist of the Wnt/β-catenin signaling pathway and can bind directly to Wnt ligands. Therefore, HBX promotes hepatocellular carcinoma by activating the Wnt/β-catenin signaling pathway through epigenetic mechanisms (Xie et al., 2014) (**Figure 1**). The activation of this pathway by HBx can result in increased expression of target genes involved in cell proliferation and tumorigenesis (Cha et al., 2004).

Furthermore, viral proteins can directly interact with cellular transcription factors and chromatin modifiers, influencing the expression of host genes (Levrero and Zucman-Rossi, 2016; Guerrieri et al., 2017; Zeisel et al., 2021). Viral proteins can recruit cellular transcriptional co-factors or chromatin-modifying enzymes to alter the chromatin structure and accessibility of specific genes (Wei and Zhou, 2010). This modulation can result in the activation or repression of genes involved in cellular growth and tumor suppression. For instance, the Epstein-Barr virus (EBV) encodes the viral protein EBNA2, which interacts with cellular transcription factors and promotes the expression of genes involved in cell proliferation. This activation of specific genes contributes to the development of EBV-associated lymphomas (Yang et al., 2021).

It is notable that viruses can also produce viral microRNAs (viral miRNAs), which target and regulate host gene expression (Skalsky and Cullen, 2010). These viral miRNAs can interfere with the expression of genes involved in cell cycle control, apoptosis, and immune responses, thereby promoting tumor formation (Bartel, 2009; Xiao and Rajewsky, 2009).It is known that Kaposi’s sarcoma-associated herpesvirus (KSHV) produces a series of miRNAs; one of them, (miR-K1) downregulated the expression of several tumor suppressor genes, including p21 (Gottwein and Cullen, 2010), leading to uncontrolled cell growth and the development of Kaposi’s sarcoma and other KSHV-associated malignancies (**Figure 1**). In addition, viral infections also affect produce of host miRNAs and microRNA dysregulation (Sartorius et al., 2021), while miRNAs often form feedback loops that regulate epigenetic modifications (Wahid et al., 2017). Furthermore, the infection causes alterations in host epigenetic modifications. Studies have shown that HBx proteins produced by HBV can initiate a variety of epigenetic modulations associated with hepatocarcinogenesis. HBx proteins can down-regulate or up-regulate the expression of some miRNAs through DNA methylation, histone methylation, and histone acetylation or HDAC inhibitors. The modification and dysregulation of host gene expression by oncogenic viruses play a critical role in tumorigenesis (Zhang et al., 2016) (**Figure 1**). By disrupting normal cellular processes, promoting uncontrolled cell proliferation, and inhibiting specific tumor suppressor, viruses create an environment conducive to the development and progression of cancer (Bouchard and Navas-Martin, 2011; Bagga and Bouchard, 2014). Understanding these mechanisms is essential for developing effective strategies to prevent or treat virus-associated cancers.

## Virus-induced host DNA methylation

Virus can induce the certain changes at DNA methylation patterns in the host genome. DNA methylation is an epigenetic modification that involves the addition of a methyl group to the DNA molecule, typically occurring at cytosine residues in CpG dinucleotides (Jin et al., 2011). It plays a crucial role in gene expression regulation, genome stability, and cellular processes (Cheung et al., 2009).

When a virus infects a host cell, it induces alterations in the host DNA methylation patterns as part of its replication and survival strategies (Di Bartolo et al., 2008; Dong et al., 2015; Cicchini et al., 2017). These changes can have significant consequences for both the virus and the host genome. Certain viruses have evolved specific mechanisms to directly manipulate host DNA methylation machinery. For example, the human immunodeficiency virus (HIV) produces a protein called viral protein R (Vpr), which can interact with and reduce the activity of DNA demethylase (TET2), leading Hypermethylation of IFTIM3 promoter (Wang and Su, 2019). This interaction can result in aberrant DNA methylation patterns in the host genome, leading to dysregulation of gene expression. Another example is that EBV in associated gastric cancer (EBVaGC) exerts epigenetic regulation of host genes. EBV causes a high frequency of methylation of DNA in the host genome e.g. several tumor suppressor genes (i.e. *APC (Adenomatous polyposis coli), PTEN (phosphatase and tensin homolog deleted on chromosome ten)*, and *RASSF1A (The Ras-Association Domain Family 1 gene, isoform A)*and cell adhesion molecules (i.e. *THBS1 and E-cadherin*) *(* Nishikawa et al., 2017) (**Figure 1**).

The host cell can initiate DNA methylation modification as a defense mechanism against viral infections (Kuss-Duerkop et al., 2018). Upon viral recognition, the host innate immune system can activate signaling pathways that promote the production of interferons and other antiviral factors (Carty et al., 2021). These activated signaling can also induce DNA methylation changes in host specific genes involved in the antiviral response, leading to their activation or repression. On the other hand, viruses exploit DNA methylation changes to evade the host immune response. Some viruses induce global DNA methylation alterations, leading to the suppression of immune response genes and the interruption of host immune defenses. By silencing immune-related genes through DNA methylation, viruses can evade immune recognition and establish persistent infections (Di Bartolo et al., 2008; Dong et al., 2015; Cicchini et al., 2017; Kuss-Duerkop et al., 2018).

Virus-induced alterations in host DNA methylation have effects on cellular gene expression patterns. Changes in DNA methylation lead to the activation or repression of specific genes, influencing various cellular processes. Viruses can exploit these changes to promote their own replication, suppress host antiviral responses, and create an environment conducive to viral survival. It is believable that Virus-induced host DNA methylation changes have been implicated in viral pathogenesis and the development of associated diseases (Hattori and Ushijima, 2016). For example, DNA methylation of a number of common host genes including *SOCS1*, *RASSF1A*, *p27*, *p21*, and *E-calmodulin* occurs in HBV-HCC. These alterations may lead to silencing of oncogenes or activation of oncogenic proteins. HBx protein can increase the transcription of DNMT1/3A/3B, resulting in the silencing of tumor suppressor genes such as *SOCS1/RASSF1A*. At the same time, it can use this mechanism to inhibit HBV cccDNA expression to evade the host immune system (Sartorius et al., 2021).The altered DNA methylation patterns disrupt normal cellular processes and promote tumor formation.

EBV infection of host cells produces a protein, latent membrane protein 1 (LMP1), LMP1 promotes the expression and activity of DNMT1, 3A and 3B, induces hypermethylation of the CDH1 promoter and down-regulation of calreticulin 1, as well as hypermethylation of the *RASSF10* tumor suppressor gene, which in turn promotes tumorigenesis (Pietropaolo et al., 2021) (**Figure 1**).

## Virus-induced host histone epigenetic modification

Virus-induced host histone epigenetic modifications also play a significant role in the development of cancer (Zhao et al., 2021). Certain oncogenic viruses have the ability to manipulate host histone modifications, thereby disrupting normal gene expression and cellular processes, contributing to tumorigenesis (Zhao and Shilatifard, 2019). Here are some key aspects of the relationship between virus-induced host histone epigenetic modifications and tumorigenesis. Oncogenic viruses modify host histone marks to activate the expression of viral oncogenes or cellular oncogenes. For instance, HPV can alter histone 3 acetylation degree and methylation patterns to activate the expression of viral oncogenes E6 and E7, which play a crucial role in HPV-associated cervical cancer development (Pietropaolo et al., 2021). Similarly, other oncogenic viruses such as Epstein-Barr virus (EBV) and Kaposi’s sarcoma-associated herpesvirus (KSHV) can manipulate host histone modifications to activate viral oncogenes and promote cellular transformation. JMJD2A is a histone demethylase of KSHV that regulates gene transactivation and cell proliferation. Its modification by small ubiquitin-like modifier (SUMO) is important for chromatin binding and viral gene expression as well as viral value addition. Blocking the SUMO-ization of JMJD2A provides a new idea in the fight against cancer (Yang et al., 2017; Okabe et al., 2020). Genome-wide analysis of 3D chromatin topologies across GC lines, primary tissue and normal gastric samples revealed chromatin domains specific to EBV-positive GC, the viral genome does not integrate into the host genome. However, it is able to cause chromatin remodeling through epigenetic modification of the host’s H3K9me3 heterochromatin by switching to H3K4me1/H3K27ac bimorph, and unleashing latent enhancers to engage and activate nearby GC-related genes (for example *TGFBR2* and *MZT1*), and then promotes gastric carcinogenesis (Okabe et al., 2020). The EBV oncoprotein LMP1 methylates the promoter of lysine-specific demethylase 2b (KDM2B), which demethylates histone 3 at the lysine 4 (H3K4me3) site. H3K4me3 is normally associated with active transcription, and demethylation results in transcriptional silencing (Vargas-Ayala et al., 2019; Pietropaolo et al., 2021).

In contrast, Virus-induced histone modifications can also lead to the silencing of tumor suppressor genes in the host genome (Fujikawa et al., 2016). Viral proteins can alter histone acetylation or methylation marks to repress the expression of tumor suppressor genes, inducing development of cancer (Khan et al., 2017). HBx proteins produced after HBV infection can induce HDAC, which in turn inhibits tumor suppressors such as *p21/p27*, an important regulator of cell cycle control, and promotes cell proliferation. In addition, HBx proteins can also induce histone methyltransferases, and it has been shown that HBx-induced up-regulation of *SMYD3*, which encodes histone H3-K4-specific methyltransferase (HMT), is related to the up-regulation of the oncogene *c-myc* in HCC. Virus-induced host histone modifications can disrupt normal cellular processes involved in cell cycle regulation (Lavia et al., 2003), DNA repair, and cell apoptosis. By altering histone marks, viruses can impact the expression of genes involved in these processes, leading to genomic instability and increased susceptibility to cancer development. Infection with HTLV-1 causes acetylation of histone H4 of the *p21^CIP1/WAF1^* coding gene. In addition, Tax and HBZ proteins produced by HTLV-1 are associated with histone acetylation. Tax can bind to CREB-binding protein (CBP) and its similar protein p300 and HDAC1, while HBZ can isolate p300/CBP, affect the interaction between Tax and p300/CBP, and cause the Tax-induced stimulation of HTLV-1 promoter to be ineffective. Because HBZ may usurp p300/CBP, it reduces the expression of cellular genes (Pietropaolo et al., 2021). The dysregulation of cellular processes by virus-induced histone modifications can contribute to the accumulation of genetic and epigenetic alterations that drive tumorigenesis. The virus-induced host histone modifications can lead to long-lasting epigenetic changes in viral-associated cancers (Jiang et al., 2020). These alterations can persist even after the clearance of the viral infection and contribute to the maintenance of malignant phenotypes. Therefore, it is important to investigate and understand these epigenetic changes, as they hold promise for the development of epigenetic-based therapies for virus-associated cancers.

## Interaction between viral proteins and host proteins

The interaction between viral proteins and host proteins plays a crucial role in tumorigenesis. Oncogenic viruses have evolved unique viral proteins that interact with host proteins to hijack cellular processes and induce tumorigenesis. Many viral proteins target host proteins involved in cell cycle regulation (Bagga and Bouchard, 2014). They interact with and modulate the activity of key regulators such as cyclins, cyclin-dependent kinases (CDKs) (Nevins, 1994; Vousden, 1995), and tumor suppressor proteins like p53 (Polager and Ginsberg, 2009) and retinoblastoma protein (pRb) (Sherr and McCormick, 2002).For instance, a novel adenoviral protein, E4-ORF3, is able to induce heterochromatin modification of H3K9me3 on the promoters of the target genes of the transcription factor p53 (also known as TP53) in a highly selective manner during virus induced carcinogenesis, thereby preventing the binding of p53 to its targets, resulting in p53 inactivation and transcriptional silencing. However, the activity of transcription factor p53 with high expression levels is important for suppressing tumorigenesis. Another virial protein E1B-55k induced p53 degradation (Soria et al., 2010). Similarly, Epstein-Barr virus nuclear antigen 3C (EBNA-3C) can directly bind to p53, to a certain extent, inhibit its transcriptional activity. The cell cycle regulatory protein E2F1, the E2F-binding protein ARID3A, and the B-cell-specific transcription factor Oct-2 bind EBNA-1, which are necessary for transcriptional activation (Yin et al., 2019). These interactions disrupt normal cell cycle checkpoints and may promote cell proliferation (Op De Beeck and Caillet-Fauquet, 1997), a hallmark of tumorigenesis.

Apart from hijacking cell cycle, viral proteins can directly interact with and inhibit the function of host tumor suppressor proteins. It has been well documented that E6 and E7 proteins of high-risk human papillomaviruses (HPVs) bind to and degrade p53 and pRb. E6 recruits the intracellular ubiquitin ligase E6AP, which is then ubiquitinated during the assembly of the E6/E6AP/p53 complex and subsequently induces proteasome-dependent p53 degradation (Li et al., 2019), E7 not only inhibits pRb activity but also promotes its degradation (Mir et al., 2023), leading to the loss of their tumor suppressor activities (Aguayo et al., 2023) (**Figure 1**). The downregulation of these tumor repressors enables uncontrolled cell growth and contributes to HPV-associated cancers, such as cervical cancer.

Host signaling pathways can be activated by viral proteins, driving cell growth and survival (Oda et al., 2022). Usually, they mimic or hijack the activity of host proteins involved in these pathways, leading to their constitutive activation (Guven-Maiorov et al., 2019). Moreover, viral proteins interfere with host proteins involved in apoptosis and immune response pathways, by which they inhibit apoptotic signals and promote cell survival, even in the presence of DNA damage or other situation cells undergoing stresses (Mesri et al., 2014). Additionally, viral proteins disrupt immune recognition and allow the cancer cells evade host immune surveillance, facilitating the persistence of the viral infection and the development of tumor-promoting inflammation.

Moreover, some viral proteins directly interact with host proteins involved in epigenetic regulation, such as DNA methyltransferases (DNMTs) and histone-modifying enzymes. The LANA protein, expressed in all Kaposi’s sarcoma-associated herpesvirus-infected cells, is able to repress host gene expression through epigenetic modification of DNA methylation. The primary mechanism is the ability of the LANA protein to interact with the DNA methyltransferase Dnmt3a in the nuclear matrix, activating Dnmt3a and transferring it to the host chromatin DNA to methylate the promoter of the down-regulated gene *cadherin 13* (*H-cadherin*) (**Figure 1**). These interactions lead to change of the enzymic activity at DNA methylation and histone modifications in host genome, as mentioned above, resulting in chromatin structure remodeling and altered gene expression involved in tumorigenesis (Shamay et al., 2006).

## Virus-induced host gene silencing

Virus-induced host gene silencing via diverse patterns (Rincon-Orozco et al., 2009). At this point, oncogenic viruses usually hijack host cellular machinery to silence specific genes that are involved in tumor suppression and regulation of cell proliferation. With unique viral proteins, virus directly or indirectly silences tumor suppressor genes that normally prevent tumorigenesis (Park et al., 2007; Jiang et al., 2021). Generally, oncogenic viruses induce epigenetic changes in the host genome, which involve DNA methylation, histone modifications, or chromatin remodeling. Viral proteins may recruit host epigenetic modifiers or interfere with the host’s epigenetic machinery to induce gene silencing (Zheng et al., 2009). Similarly, the core proteins of HCV capable of inducing HCC can lead to epigenetic silencing of host tumor-suppressor genes by regulating host levels of DNA methyltransferases. As another example, HTLV-I infection leads to reduced hyperacetylation of the host CD3 histone H4, which in turn leads to its reduced expression. The absence of TCR/CD3 expression then contributes to malignancy (Akl et al., 2007) (**Figure 1**).

Some silenced genes by viruses may be involved in host signaling pathways by targeting key components of signaling cascades, viruses disrupt the normal balance and regulation of cell growth and survival. Skalska et al. reported that Epstein-Barr virus (EBV) can silence genes involved in the p16INK4a-Rb pathway, leading to dysregulation of cell cycle control and contributing to the development of EBV-associated lymphomas (Ohtani et al., 2003).In addition, by suppressing the expression of immune-related genes, viruses inhibit host response against infected or tumorigenesis. This immune evasion strategy allows viruses to persist in the host and/or promote tumorigenesis. Through silencing genes that are crucial for these processes, viruses can promote genomic instability, inhibit programmed cell death, and interfere with cellular differentiation, contributing to the development of cancer.

## Virus-induced host gene activation

Apart from gene silencing, certain viruses can also induce the activation or overexpression of host genes, thereby facilitating cancer development. Oncogenic viruses directly activate host proto-oncogenes, which are necessary cellular genes that, when mutated or overexpressed, may contribute to cancer development. In mechanism, viral proteins interact with regulatory elements or transcription factors of these genes, leading to their enhanced expression.

Human T-cell leukemia virus type 1 (HTLV-1) produces a Tax protein, which activates cellular proto-oncogenes such as c-Myc and promotes the development of adult T-cell leukemia/lymphoma (Duyao et al., 1992). Similarly, in the Burkitt lymphoma cells, bromodomain-containing protein 7 is hijacked by EBV to synergistically maintain viral latency through its encoded EBNA1 binding to host BRD7 to coordinate chromatin remodeling, leading to transcriptional activation of c-Myc. EBV-mediated BRD7 was enriched around in the enhancer in the lgH locus (Andersson et al., 2014), which might activate the c-Myc alleles. If BRD7 is disrupted, the expression of c-Myc will decrease and the virus will enter the lytic phase (Li et al., 2023). Moreover, viral proteins activate host gene expression via intervening with host signaling pathways. Through interacting with key components of these pathways, viruses activate downstream signaling cascades, recruiting specific transcript complex to promoters of the target genes, resulting in an enhanced expression. Hepatitis C virus (HCV) protein NS5A, for instance, can activate the PI3K-Akt signaling pathway, which promotes cell survival and contributes to hepatocellular carcinoma (HCC) development (Cheng et al., 2015; Raja et al., 2017; Raja et al., 2022).

In transcription level, viral proteins interact with host transcriptional regulators and modify their activity, leading to the activation of specific host genes involved in tumorigenesis. These interactions usually occur through direct protein-protein interactions. For example, the Epstein-Barr virus (EBV) protein EBNA2 interacts with host transcription factors such as RBP-Jκ(by interfering with the function of the co-repressors and providing activating structural domains)and activates expression of the genes involved in B-cell growth and survival, contributing to the development of EBV-associated lymphomas (Waltzer et al., 1995) (**Figure 1**).

Genome stability is required for normal cellular processes, viruses interfere with host DNA repair mechanisms, resulting in genomic instability and the activation of oncogenes. The human papillomavirus (HPV) proteins E6 and E7, disrupt DNA repair processes, leading to the accumulation of DNA damage and the activation of cellular oncogenes (Bruyere et al., 2023). Otherwise, some viruses can indirectly induce the production of growth factors and cytokines through infecting host cells. These factors promote target gene expression involved in cell proliferation and survival, creating a condition conducive to tumorigenesis. Kaposi’s sarcoma-associated herpesvirus (KSHV) produces viral interleukin-6 (vIL-6), which stimulates the proliferation of infected cells and contributes to the development of Kaposi’ssarcoma (Wu et al., 2014). In conclusion, different viral components activate regulators of gene expression via diversity mechanism in host cells, leading to abnormal gene expression and resulting in tumor development.

## Perspective

Growing evidence strongly suggests a significant relationship between viral infections and the development of tumors. The study of virus-induced alterations in host gene expression within cancer offers a unique perspective on understanding the intricate interplay between viral infections and oncogenesis. By examining changes in gene expression patterns induced by viruses, researchers can uncover novel pathways and mechanisms involved in cancer development. However, due to the diversity of viruses and the complex regulation of host gene expression, scientists must identify various viral proteins implicated in host genome remodeling and/or tumorigenesis. Simultaneously, we need to elucidate the host proteins that interact with viral proteins and the downstream pathways mediated by these interactions.

In this review, we have endeavored to provide valuable insights into the molecular basis of virus-associated cancers, potentially leading to the identification of new intervention and therapeutic targets, as well as the development of innovative treatment strategies. Furthermore, our work deepens our understanding of host-virus interactions and the complex molecular events driving tumorigenesis, paving the way for improved diagnostic and prognostic approaches in cancer management for the future.”

## Author contributions

WD: Formal Analysis, Methodology, Resources, Software, Writing – original draft. HW: Methodology, Writing – original draft, Conceptualization, Investigation, Supervision. ML: Conceptualization, Resources, Validation, Writing – original draft. PL: Writing – original draft, Data curation, Methodology, Software. SJ: Funding acquisition, Supervision, Validation, Writing – review & editing.
